# Matching curated genome databases: a non trivial task

**DOI:** 10.1186/1471-2164-9-501

**Published:** 2008-10-24

**Authors:** Stéphane Descorps-Declère, Matthieu Barba, Bernard Labedan

**Affiliations:** 1 Institut de Génétique et Microbiologie, Université Paris Sud XI, CNRS UMR 8621, Bât. 400, 91405 Orsay Cedex, France

## Abstract

**Background:**

Curated databases of completely sequenced genomes have been designed independently at the NCBI (RefSeq) and EBI (Genome Reviews) to cope with non-standard annotation found in the version of the sequenced genome that has been published by databanks GenBank/EMBL/DDBJ. These curation attempts were expected to review the annotations and to improve their pertinence when using them to annotate newly released genome sequences by homology to previously annotated genomes. However, we observed that such an uncoordinated effort has two unwanted consequences. First, it is not trivial to map the protein identifiers of the same sequence in both databases. Secondly, the two reannotated versions of the same genome differ at the level of their structural annotation.

**Results:**

Here, we propose CorBank, a program devised to provide cross-referencing protein identifiers no matter what the level of identity is found between their matching sequences. Approximately 98% of the 1,983,258 amino acid sequences are matching, allowing instantaneous retrieval of their respective cross-references. CorBank further allows detecting any differences between the independently curated versions of the same genome. We found that the RefSeq and Genome Reviews versions are perfectly matching for only 50 of the 641 complete genomes we have analyzed. In all other cases there are differences occurring at the level of the coding sequence (CDS), and/or in the total number of CDS in the respective version of the same genome.

CorBank is freely accessible at . The CorBank site contains also updated publication of the exhaustive results obtained by comparing RefSeq and Genome Reviews versions of each genome. Accordingly, this web site allows easy search of cross-references between RefSeq, Genome Reviews, and UniProt, for either a single CDS or a whole replicon.

**Conclusion:**

CorBank is very efficient in rapid detection of the numerous differences existing between RefSeq and Genome Reviews versions of the same curated genome. Although such differences are acceptable as reflecting different views, we suggest that curators of both genome databases could help reducing further divergence by agreeing on a minimal dialogue and attempting to publish the point of view of the other database whenever it is technically possible.

## Background

Public genomic databanks are inexorably inundated by newly sequenced genomes. The number of complete sequence of prokaryotic genomes that are published per year has increased more than tenfold in the last seven years with a present rate close to four newly published prokaryotic genomes per week. One of the main challenges encountered by genome databanks is that complete genomic sequences are submitted with a heterogeneous and (too) often crude gene annotation [1-4]. To cope with these major problems and to improve the representation of genomic information, NCBI and EBI are proposing curated versions, the Reference Sequence (RefSeq) [5] and Genome Reviews [6], respectively. Each database team is working independently but they share the same main goal of delivering an up-to-date, standardized and comprehensive view of the completely sequenced genomes that are present in the International Nucleotide Sequence Database (INSD) repository (GenBank/EMBL/DDBJ),

To facilitate the use of these standardized genomic data in comparative genomics studies, both RefSeq and Genome Reviews include manually curated information. Noticeably, RefSeq and Genome Reviews provide cross-references to public databases to facilitate database searches. Interestingly, many of these cross-references (/db_xref) are specific to the curated database: for instance, RefSeq has/db_xref to Entrez [7] and often to CDD [8], whereas Genome Reviews has/db_xref to Gene Ontology [9], InterPro [10], and UniProt [11], and occasionally to HOGENOM [12], and PDB [13].

Thus, it would be advantageous to work with both curated databases since they look more complementary than concurrent. However, there is no immediate way to match the respective sequence identifiers listed by either RefSeq or Genome Reviews for the same gene of the same reannotated genome, although the knowledgebase UniProt [11] began to add links to both genome databases as this paper was in preparation. Moreover, the independent efforts of NCBI and EBI curators in improving the structural annotation of a few CDS, lead to increasingly different genomic versions of the same organism. Three different instances are expected when comparing the structural annotations made independently by RefSeq and Genome Reviews curators: (i) the amino acid sequences are exactly identical, (ii) both CDS share an overlapping identical segment but differ in length, (iii) a few CDS are found exclusively in one genome database. This last instance corresponds often to the redefinition of a putative CDS as being a pseudogene on the basis of structural features.

We aimed to obtain immediate and exhaustive cross-references of each protein-coding gene when dealing with such possible divergences that reflect different points of view between RefSeq and Genome Reviews. Accordingly, we designed CorBank, a software (see [14]) that detects not only perfect identities but also any differences between RefSeq and Genome Reviews databases.

## Results

**Figure 1:**
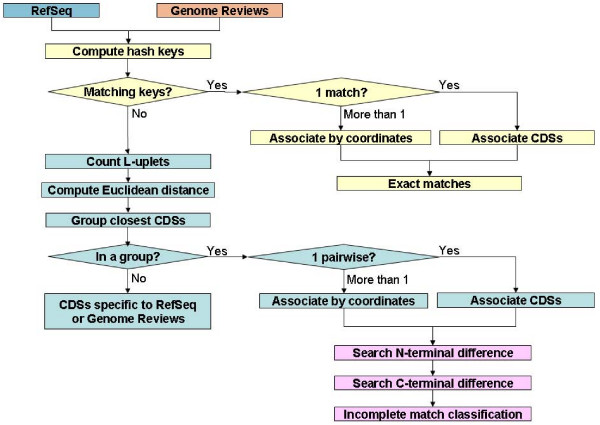
**The different steps of the CorBank program**. The main steps of the pipeline of Perl scripts are distinguished by different colors. The process of cross-referencing exact matching of the RefSeq and Genome Reviews versions of the same gene is indicated in yellow. The identification of inexact matches of genes that display a different structural annotation in both databases is made by the blue steps. Finally, disclosing the nature of the detected structural differences is made by the pink steps.

Complete sequences of each replicon of each prokaryotic organism endowed with the same Taxonomy ID in both RefSeq and Genome Reviews were downloaded from each database and mapped by their common INSD identification numbers. Then, as schematized on Fig. 1, we compared both database versions of the same genomic data to identify the cross-references for each gene and to measure their level of matching. Accordingly, the different scripts that make up the CorBank program [14] were applied to these mapped data in two successive steps in order first to find exact matches and then to identify the nature and location of any difference in imperfect matches.

### Matching gene sequences in independently curated genome databases

To be as fast as possible, we did not compare the sequence partners by using efficient but slow programs such as BLASTClust [15]. Rather, we used the Perl language to build hash tables where each amino acid sequence is a key that indexes its encoding CDS. Matching is straightforward when the same key is found for the two versions of the same gene sequence – one in RefSeq and the other in Genome Reviews (Fig. 1, yellow part). In rare instances, more than two identical sequences were found for the two versions of the same genome. This occurred for example with strictly identical insertion sequences present at different locations on the analyzed genome. Moreover, we could not dismiss the hypothesis that in very very rare cases pairs of completely conserved paralogues could form bidirectional best matches that may be erroneously interpreted. To handle these problems, we further used the respective gene positions to identify the pertinent couples of corresponding sequences (Fig. 1, yellow part).

**Figure 2:**
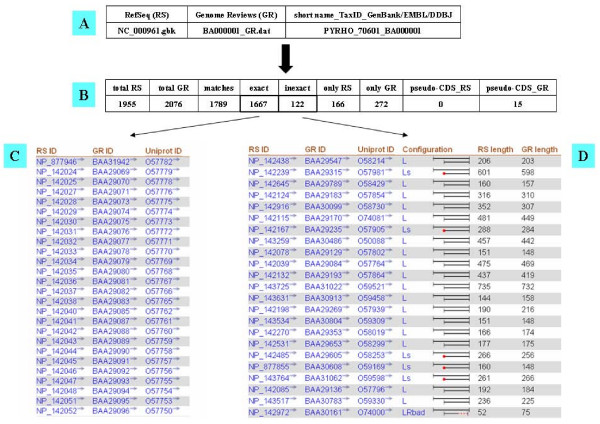
**Differentiating exact and inexact matches**. A partial view of the output of the CorBank program obtained when comparing the two versions of the genome of the archeon *Pyrococcus horikoshii *OT3 is detailed in several tables. Table A recapitulates the respective database information about this species and its computed label. Table B shows a summary of the data obtained using CorBank to find what is either common to both databases or specific of each one. Table C illustrates a few instances of exact matches. Table D exemplifies a few inexact matches with detailed configuration of the difference in the structural annotations of each copy of the same gene. The definitions of these inexact configurations are given in the Additional file 1.

Using this approach based on hash tables, we found that 98% of copies of the 1,983,258 genes described in both databases are matching, allowing instantaneous retrieval of their respective cross-references (see, for instance, Fig. 2 Table C).

However, the view was more contrasted when comparing complete genome annotation instead of looking at each individual gene. Table 1 shows that only 50 of the 641 complete genomes we have analyzed are perfectly matching at the level of their structural annotation. The other ones differ in terms of their respective total number of sequences and/or distribution of perfect matching sequences (Table 1). The copies in both curated databases of 260 genomes differ by their total numbers of genes and by a significant proportion (up to 12.5%, see below *Xanthomonas oryzae pv. oryzae KACC10331 *in Table 3) of inexact matching of individual genes. The two versions of 321 species differ by their respective total numbers of genes but their corresponding CDS are matching exactly. For instance, *Bordetella petrii DSM 12804 *has 5004 CDS that are matching exactly but RefSeq contains 23 CDS that are absent from Genome Reviews, whereas Genome Reviews display four additional CDS and 24 pseudo-CDS (amino acid sequence without a protein_id) that are not present in the RefSeq file. Finally, only 10 genomes have the same total numbers of genes but up to 7.4% of their corresponding genes display inexact matching. For instance, *Xanthomonas campestris pv. campestris str. 8004 *displays 4273 CDS in both genomic databases but the respective amino acid sequence of the product of 310 of them differ between RefSeq and Genome Reviews. Complete data are available in Additional file 1 and on the CorBank site [14]).

**Table 1:** The reannotated copies of the same genome in independently curated databases^a ^are predominantly divergent

**copies of the same genome sequence in both curated databases^a ^with**		**all CDS matching exactly**	
		
		*NO*	*YES*
**identical number of genes**	*NO*	260 (40.5%)	321 (50%)
	*YES*	10 (1.5%)	50 (8%)

^a ^RefSeq (Release 30) and Genome Reviews (Release 94.0) of July 2008

### Defining peculiarities of gene sequences that are partially identical between independently curated genome databases

We further studied these imperfectly matching sequences by measuring their similarity using an alignment-free approach (for a review and references inside, see [16]). Indeed, such an approach is fast and well-adapted to comparison of varying versions of the same sequence that share a significant common part. As detailed in Methods, we calculated the Euclidean distance that separates the distributions of words of length *L *(= 10) for each copy of the same gene in RefSeq and Genome Reviews, respectively (Fig. 1, blue part). This allows finding the cross-references between the respective imperfectly matching copies of the same gene (see, for instance, Fig. 2 Table D). A large variety of differences explaining these imperfect matches have been found using the CorBank program (Fig. 1, pink part). All of these differences – including the very rare ones – have been categorized as summarized in the Additional file 1 and on the page . CorBank is able to filter any differences in any sequence locations (see, for instance, Fig. 2 Table D).

We found that the differences between matching sequences that have unequal lengths were predominantly (98.7%) located at the N-terminal part. Indeed, it is often difficult to identify the start codon, especially when several methionines are found in this N-terminal region (see, for example, [17]).

### Identifying the whole differences separating independently curated copies of a genome

**Figure 3:**
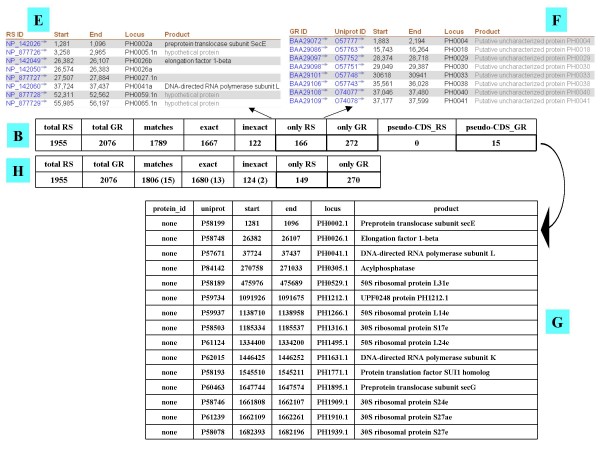
**Differentiating exact and inexact matches, following**. Table E illustrates a few instances of genes found uniquely in RefSeq. Table F exemplifies a few genes specific to Genome Reviews. Table G lists the pseudo-CDS specific to Genome Reviews. Table H re-evaluates the data presented in Table B after identifying by their positions the pseudogenes and pseudo-CDS specific to RefSeq and Genome Reviews, respectively and assessing their exactitude.

Scanning paired versions of the same genome with CorBank allows computing the statistics of similarities and differences between genome databases. Figs. 2 and 3 detail the results obtained with the archaeon *Pyrococcus horikoshii*. The genomes of three *Pyrococcus *have been published ten years ago: *P. horikoshii *in 1998 [18], *P. abyssi *in 1999 [19] and *P. furiosus *in 2000 [20]. Since then, these genomes, sequenced and annotated by independent groups, have been curated several times. Fig. 2 shows that many differences have accumulated between the curated versions of the *P. horikoshii *genome in RefSeq and Genome Reviews (Fig. 2 Table B). First, the respective total numbers of genes are strikingly different. Among the 1955 sequences published in RefSeq and the 2076 ones listed in Genome Reviews, only 1789 are matching. Secondly, we have only 1667 of these matches that are exact (Fig. 2 Table C), while 122 display various differences. Fig. 2 (Table D) details a few instances of these differences in length and location of the start and end of each gene. Thirdly, Fig. 3 shows that there are a significant number of sequences putatively encoded by the *P. horikoshii *genome that are found in uniquely one genome database: 166 genes in RefSeq (Fig. 3 Table E) and 272 in Genome Reviews (Fig. 3 Table F), respectively. However, Genome Reviews classifies as pseudo-CDS a list of 15 amino acid sequences which have no protein_id. Since these pseudo-CDS are found as standard coding sequences among the 166 sequences that are specific to RefSeq (Fig. 3 Table G), we ascertained this point. CorBank was further used to match these 15 pseudo-CDS using uniquely the position information that have been kept in both databases. As a result, Fig. 3 Table B was improved in Fig. 3 Table H after matching 13 of the 15 Genome Reviews pseudo-CDS as exact and two ones as inexact. Thus, it appears that RefSeq and Genome Reviews are producing increasingly divergent views of the same genome.

Table 2 shows the same trend for the two other *Pyrococcus *species, although the divergence is less marked. Such a discrepancy is strongly diminished when looking at the genome of the related *Thermococcus kodakarensis*, belonging to the same family (Thermococcaceae), which has been published more recently (in 2004 [21]). However, this example does not reflect a general (statistical) trend between the amount of divergences and time elapsed since the completion of sequence that would be true for all analyzed genomes (see below Tables 3 and 4 and accompanying text).

**Table 2:** Complete distributions of the divergences of curated databases^a ^in the case of closely related species

**analyzed species**	**Comparing CDS in RefSeq Release 30 (RS) and Genome Reviews Release 94.0 (GR) databases^a^**							
	
	***total number***		***matches***				***specific to***	
	***RS***	***GR***	***total***	***exact***	***inexact***	***by location***	***RS***	***GR***
P. horikoshii	1955	2076	1806	1680	124	0	149	270
P. furiosus	2125	2065	2065	1942	115	8	60	0
P. abyssi	1896	1786	1783	1715	68	0	113	3
T. kodakarensis	2306	2306	2306	2303	2	1	0	0

^a ^versions of May 2008

**Table 3:** Top ten organisms having the highest number of CDS specific to RefSeq (RS) database

**rank**	**organism**	**Total**		**matches**			**specific to**	
		
		***RS***	***GR***	***total***	***inexact***	***by location***	***RS***	***GR***
RS1/GR7	Pyrococcus horikoshii OT3	1955	2076	1806	124	0	**149**	270
RS2	Neisseria meningitidis Z2491	2049	1991	1897	37	26	**120**	68
RS3/GR4	Xanthomonas oryzae pv. oryzae KACC10331	4144	4540	4030	497	2	**114**	510
RS4	Pyrococcus abyssi GE5	1896	1796	1783	68	0	**113**	3
RS5/GR6	Shewanella oneidensis MR-1	4467	4779	4364	34	1	**103**	415
RS6/GR8	Escherichia coli O157:H7 str. Sakai	5318	5461	5227	391	2	**87**	232
RS7	Deinococcus radiodurans R1	3181	1303	3099	91	1	**82**	4
RS8	Pyrococcus furiosus DSM 3638	2125	2065	2065	115	8	**60**	0
RS9	Lactococcus lactis subsp. lactis Il1403	2321	2266	2263	68	0	**58**	3
RS10	Thermoplasma volcanium GSS1	1499	1526	1444	351	1	**55**	82

The organisms are sorted by their respective rank that is computed as the number of CDS that are found only in RefSeq database (Release 30). The organism names standing in the top ten list of both databases (Tables 3 and 4) are in bold.

**Table 4:** Top ten organisms having the highest number of CDS specific to Genome Reviews (GR) database

**rank**	**organism**	**Total**		**matches**			**without sequence or specific to**	
		
		***RS***	***GR***	***total***	***inexact***	***by location***	***RS***	***GR***
GR1	Mycobacterium leprae TN	1605	2723	1605	77	1	0	1118
GR2	Orientia tsutsugamushi str. Boryong (Seoul National University)	1182	2143	1182	3	0	0	961
GR3	Orientia tsutsugamushi str. Boryong (Kitasato University)	1562	2085	1562	6	0	0	523
GR4/RS3	Xanthomonas oryzae pv. oryzae KACC10331	4144	4540	4030	497	2	114	510
GR5	Acinetobacter baumannii ATCC 17978	3368	3807	3368	77	0	0	439
GR6/RS5	Shewanella oneidensis MR-1	4467	4779	4364	34	1	103	415
GR7/RS1	Pyrococcus horikoshii OT3	1955	2076	1806	124	0	149	270
GR8/RS6	Escherichia coli O157:H7 str. Sakai	5318	5461	5227	391	2	87	232
GR9	Prochlorococcus marinus subsp. pastoris str. CCMP1986	1717	1935	1714	4	2	3	221
GR10	Prochlorococcus marinus str. MIT 9312	1810	1962	1810	10	0	0	152

The organisms are sorted by their respective rank that is computed as the number of CDS that are found only in Genome Reviews database (Release 94.0). The organism names standing in the top ten list of both databases (Tables 3 and 4) are in bold.

## Discussion

CorBank is fulfilling two complementary goals: (1) to deliver immediate cross-references between each copy of each gene published in both RefSeq and Genome Reviews genome databases; (2) to identify any differences between both independently curated structural annotations. The first objective is achieved almost immediately: e.g. cross-referencing the two databases versions of a 3000 CDS genome is completed in less than 1 second on a basic home computer. Exhaustive comparison of the 641 prokaryotic species present in both databases at the end of July 2008 (Genome Reviews Release 94.0, 22nd July 2008 – RefSeq Release 30, July 11, 2008) has been completed in less than 60 min. Thus, the efficiency of CorBank is largely equivalent to that of the PICR tool that is described in a paper [22] that appeared as we were writing a first version of this manuscript. PICR, a web service allowing matching a large variety of protein sequence identifiers, is restricted to 100% identity matches and cannot discriminate the correct pair when recovering more than two identical sequences since it does not exploit information about genomic locations, contrarily to Corbank. Thus, this PICR tool and a previous one, MagicMatch [23], are not as efficient as CorBank to match exhaustively genome databases. This quality is especially true of our second goal that is achieved uniquely by CorBank. Its exhaustive comparison of the species currently present in both RefSeq and Genome Reviews shows dramatic differences in the structural annotations of a large portion of their copies of the same genomes (Tables 1 to 3, Figs. 2 and 3). Of the 641 compared genomes, 581 differ in their total numbers of CDS and 270 have from 1 to 781 coding sequences per genome that differ in length.

The large majority of the 50 perfectly matching genomes correspond to newly sequenced species where the manual curation has not been started. However, there is no direct correlation between the sequencing age and the level of divergence between the lastly curated versions of the same genome as shown on Tables 3 and 4 that list the top ten database-specific organisms in both RefSeq and Genome Reviews, respectively. Actually, a Spearman test failed to show any correlation of the different parameters computed by CorBank with the time elapsed since the completion of sequence (not shown).

Surprisingly, even the two versions of a model organism such as *Escherichia coli *K12 (substrain MG1655) that has been recently extensively reannotated in cooperative works [24,25] display significant differences. Of the 4295 gene-encoding proteins, only 4130 are matching (including 10 inexact matches), and both databases differ in their interpretation of some genes as being described as pseudo-CDS: 23 in RefSeq versus 24 in Genome Reviews. In fact, the structural identification of putative pseudogenes in *E. coli *K12 has been previously described (see [26] and references inside) but it is surprising that there is still disagreement even for these *E. coli *K12 pseudogenes.

As we were writing this paper, UniProtKB began to add/db_xref to RefSeq and Genome Reviews protein_id. However, we observed that rather often the same SwissProt file has cross-references to multiple RefSeq and Genome Reviews protein_id. This is why we think that CorBank is – presently – the only software publishing unambiguous mapping of RefSeq, Genome Reviews, and UniProt identifiers of a protein.

## Conclusion

Data dependencies inherent to the annotation process by homology make genome data predestined for propagated errors [1-4]. Thus, data cleansing is a necessity for genome data after the data is produced. However, such cleansing is uneasy since it is often impossible to find the correct solution right away. Instead, there often exists a set of alternative solutions. Accordingly, RefSeq and Genome Reviews appear to have diverged in looking for correct solutions when performing credibility checking on the INSD crude data. Credibility checking is a very important step for genome data production since the correctness of data is crucial before it is used within other processes such as annotation of newly sequenced genomes by homology to previously annotated genomes. However, such independent efforts made by both automatic and manual procedures [5,6] led to increasingly divergent reannotated data as shown in this work. Clearly, the time has come to enable curators of both genome databases to establish a minimum of dialogue. Whenever it would be technically possible, a useful compromise may be found where each database publishes the point of view of the other one. We acknowledge that such a harmonization effort looks rather complicated to be done. However, it would be very helpful for the whole community.

## Methods

### Comparing copies of the same genomes in curated databases

The whole genomic sequences present in RefSeq [5] and Genome Reviews [6] were downloaded at their respective FTP sites [27,28]. A first script allows matching respective downloaded files for the same genome. This script creates a mapping list between the replicons (chromosomes and plasmids) of the genome databases RefSeq and Genome Reviews. It links each respective genome identifier by using their common INSD identifier. A recognizable label, based on the association of its short name, NCBI tax_id and its INSD identifier, is associated to each matched replicon, e.g. PYRHO_70601_BA000001 for *Pyrococcus horikoshii *OT3 [17]. CorBank further compiles for each analyzed species the respective number of perfect and imperfect matches, and the sequences that are specific to a genome database as detailed below and in Fig. 1.

### Detecting perfect matches between copies of the same gene in RefSeq and Genome Reviews

We built hash tables where each amino acid sequence is a key that indexes its encoding CDS (Fig. 1, yellow part). Each time the same key is found for the two versions of the same gene sequence made possible to cross-reference the respective protein identifiers in RefSeq [5], Genome Reviews [6], and UniProt [11] as shown on Fig. 2 (Table C).

### Estimating similarity of partially identical sequences

In a second step (Fig. 1, blue part), CorBank is detecting all imperfect matches using an alignment-free comparison [for a review, see [16]]. We used a word approach as initially proposed by Blaisdell [29] and further documented by Zharkikh and Rzhetsky [30] to measure the similarity between sequences without any alignment. The distribution of the frequency of words of length *L *(*L *= 10 residues) in each amino acid sequence was computed for both copies of the same gene. These *L-uplets *are the respective signature of the sequence. The measure of the similarity between both copies of the same sequence is based on the Euclidean distance *d*^*E *^that separates them:

$$d_{L}^{E}(X,Y)=\sum_{i=1}^{K}{\left(c_{L,i}^{X}-c_{L,i}^{Y}\right)}^{2}$$

The vectors $c_{L}^{X}$ and $c_{L}^{Y}$ represent word counts for the versions *X *and *Y *of the amino acid sequences encoded by the same gene in the respective RefSeq and Genome Reviews versions and K is the number of different *L- uplets *possible for the *L*-length. These *X *and *Y *copies are expected to share a largely common part but are of unequal sizes, one copy having an extension of variable size. To exclude any bias due to too large extensions, we stated that the maximum value of the distance *d *that separates two unequal copies of the same sequence could not be less than the difference between their respective numbers of amino acids.

In a third step (Fig. 1, pink part), CorBank is further analyzing all imperfect matches to define the location of the difference between both paired copies of the same gene. CorBank is first searching if the difference takes place on either the N-terminal side or the C- terminal one. In rare cases, the difference is located elsewhere, including in the common segment of both copies that could differ for only one residue. The Additional file 1 details all encountered cases, including the very rare ones.

## Authors' contributions

SDD inspired using a word approach. SDD and MB developed together the CorBank program. MB set up the present CorBank website and also made in-depth analysis of the data obtained with CorBank. BL initiated the work, participated in the data analysis and wrote the draft manuscript. All authors read and finalized the whole version of the manuscript.
